# Elevation of intracranial pressure affects the relationship between hemoglobin concentration and neuronal activation in human somatosensory cortex

**DOI:** 10.1002/hbm.24973

**Published:** 2020-03-04

**Authors:** Julia Thranitz, Martin Knauth, Marcus Heldmann, Jan Küchler, Thomas F. Münte, Georg Royl

**Affiliations:** ^1^ Department of Neurology, Center of Brain, Behavior and Metabolism University of Lübeck Lübeck Germany; ^2^ Department of Internal Medicine Schön Klinik Neustadt Neustadt in Holstein Germany; ^3^ Department of Neurosurgery University of Lübeck Lübeck Germany

**Keywords:** BOLD‐fMRI, brain mapping, functional neuroimaging, intracranial pressure, near‐infrared spectroscopy, neurovascular coupling, somatosensory evoked potentials

## Abstract

During neuronal activation, a local decrease of deoxygenated hemoglobin concentration (deoxy‐Hb) occurs which is the basis of functional brain imaging with blood oxygenation level dependent functional magnetic resonance imaging (BOLD‐fMRI). Elevated intracranial pressure (eICP) has been shown to impair functional deoxy‐Hb changes. This study investigated this effect and its relation to the underlying neuronal activity in the human primary somatosensory cortex (SI). Functional near‐infrared spectroscopy (fNIRS) during somatosensory evoked potentials (SEP) monitoring was performed on 75 subjects during conditions of median nerve stimulation (MNS) and resting state, combined with normal breathing (NB) and eICP by escalating breathing maneuvers (breath holding [BH], Valsalva maneuver with 15 mmHg [V15] and 35 mmHg expiratory pressure [V35]). During NB, fNIRS revealed a typical oxygenated hemoglobin concentration (oxy‐Hb) increase with deoxy‐Hb decrease during MNS enabling SI brain mapping. Breathing maneuvers associated eICP produced a known global change of oxy‐Hb and deoxy‐Hb with and without MNS. When subtracting measurements during resting state from measurements during MNS, neither functional oxy‐Hb nor deoxy‐Hb changes could be recovered while SEPs remained unchanged. In conclusion, Valsalva‐induced eICP prevents oxy‐Hb and deoxy‐Hb changes during neuronal activation in SI. This finding raises questions on the validity of oxy‐Hb‐ and deoxy‐Hb‐based brain imaging (e.g., BOLD‐fMRI) during eICP.

**Abbreviations**
BH: breath holding without Valsalva maneuver
BOLD: blood oxygenation level dependent
CBV: cerebral blood volume
CSF: cerebrospinal fluid
deoxy‐Hb: deoxygenated hemoglobin concentration
EEG: electroencephalography
eICP: elevated intracranial pressure
fMRI: functional magnetic resonance imaging
fNIRS: functional near‐infrared spectroscopy
Hb: hemoglobin
ICP: intracranial pressure
MI: primary motor cortex
MNS: median nerve stimulation
NB: normal breathing
NVC: neurovascular coupling
oxy‐Hb: oxygenated hemoglobin concentration
PetCO_**2**_: partial pressure of end‐tidal carbon dioxide
SEP: somatosensory evoked potential
SI: primary somatosensory cortex
SNR: signal to noise ratio
total‐Hb: total hemoglobin concentration
V15: Valsalva maneuver with 15 mmHg forced expiratory pressure against resistance
V35: Valsalva maneuver with 35 mmHg forced expiratory pressure against resistance
VM: Valsalva maneuver

## 1 INTRODUCTION

Monitoring vascular responses to detect local brain activation forms the basis of numerous important neuroimaging methods (e.g., functional magnetic resonance imaging (fMRI), functional near‐infrared spectroscopy (fNIRS), positron emission tomography, single‐photon emission computed tomography, intrinsic signal optical imaging) that have been established in neuroscience studies (Villringer & Dirnagl, 1995). Although known for years (Roy & Sherrington, 1890), the origin, purpose, underlying mechanisms, and influencing factors of neurovascular coupling (NVC) have not been clarified in detail (Hillman, 2014; Iadecola, 2017; Leithner & Royl, 2014; Masamoto, Hirase, Yamada, & Kanno, 2016; Mathias, Plank, & David, 2017). This raises issues on the validity of blood oxygenation level dependent (BOLD)‐fMRI which has been advanced to clinical routine, for example in presurgical brain tumor mapping (Pak et al., 2017; Wang et al., 2012), since it is based on the concentration of deoxygenated hemoglobin (deoxy‐Hb) rather than neuronal activity. Similarly, notwithstanding this limitation, fNIRS is also being adapted to patient applications, for example, in brain‐computer‐interfacing (Banville, Gupta, & Falk, 2017). A recent review has put forward the problem that on the one hand observed oxygenation changes in fNIRS signals can be falsely attributed to neuronal activity although their origin is systemic or even extra‐cerebral, and on the other hand fNIRS can miss neuronal activity because systemic reactions interfere with oxygenation changes (Tachtsidis & Scholkmann, 2016).

Elevated intracranial pressure (eICP), a condition often associated with brain tumors, has been found to relevantly impair NVC. In a rat model, eICP caused by mock cerebrospinal fluid (CSF) infusion led to an amplitude reduction and—during very high intracranial pressure (ICP)—a reversal of deoxy‐Hb changes during primary somatosensory cortex (SI) activation (Füchtemeier et al., 2010). We recently reproduced this finding in human primary motor cortex (MI) when applying Valsalva‐induced eICP during MI activation via finger tapping and finding an amplitude decrease of functional deoxy‐Hb changes ultimately leading to a complete abolishment of functional brain imaging with fNIRS when based on deoxy‐Hb (Knauth, Heldmann, Münte, & Royl, 2017). In order to reproduce this effect in a different cortical area, and at the same time enabling the obtainment of a direct, that is, electrophysiological, measurement of concurrent neuronal activation we transferred this study to human SI by investigating neuronal activation and NVC responses during median nerve stimulation (MNS) with different breathing maneuvers leading to changes in ICP.

## 2 MATERIALS AND METHODS

The study was approved by the ethical committee at the University of Lübeck and conducted in accordance with the Declaration of Helsinki (64. WMA General Assembly, October 2013, Fortaleza, Brazil) and the understanding and informed consent of each subject prior to their inclusion in the study.

### 2.1 Subjects

Seventy‐five healthy volunteers (mean age 23.5 years; range 18–47 years; 47 women; 66 right‐handers) with no history of neurological or pneumological disorders participated in the study.

### 2.2 Electrophysiological measurement

In order to obtain a direct measurement of neuronal activity during MNS, somatosensory evoked potentials (SEP) were recorded as applied in clinical routine. Two EEG electrodes were placed on the subject's scalp at the C3′ = CP3 (right‐hand stimulation) and C4′ = CP4 (left‐hand stimulation) position above the postcentral gyrus, 3 cm occipital and 7 cm lateral of Cz, according to the 10–20 system for EEG (Nuwer et al., 1998) and fixed with 3 × 3 cm white plaster. A reference electrode was placed on the forehead and a ground electrode a few centimeters proximal of the stimulation block above the carpal tunnel. Impedances were kept below 5 kΩ. The electrodes were connected to the measuring unit of the applied ISIS IOM System (OSIRIS Neurostimulator, NeuroExplorer software version 4.4.9.0, INOMED, Emmendingen Germany) that also triggered MNS. SEPs were averaged within each trial and recorded to the hard disk of the connected computer.

### 2.3 fNIRS measurement

**Figure 1:**
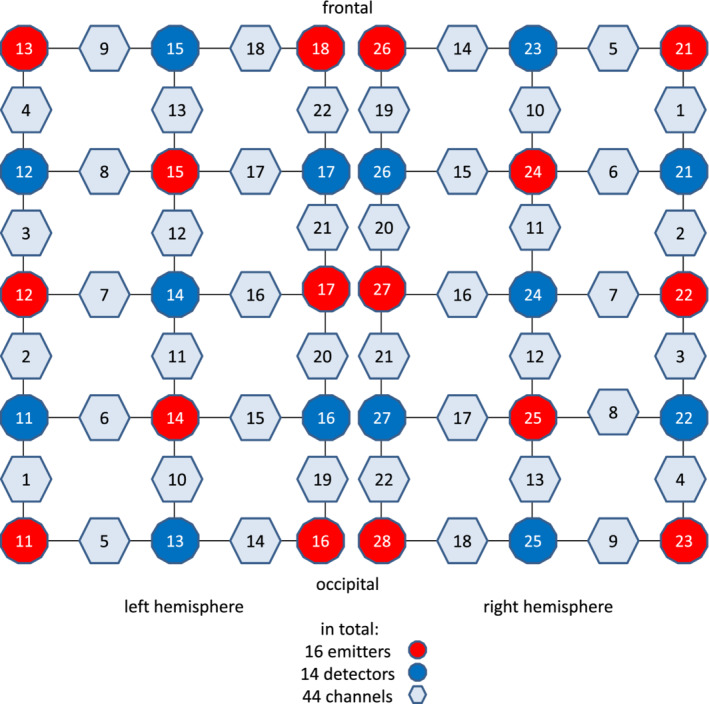
Optode grid. A total of 30 optodes (16 emitters alternating with 14 detectors) was attached to an elastic head cap and arranged as two 3 × 5 grids with an area of 14 × 19 cm each covering the left and right hemisphere's SI. From these 2 × 15 optodes 2 × 22 detector channels were generated, located halfway between emitter and detector

**Figure 2:**
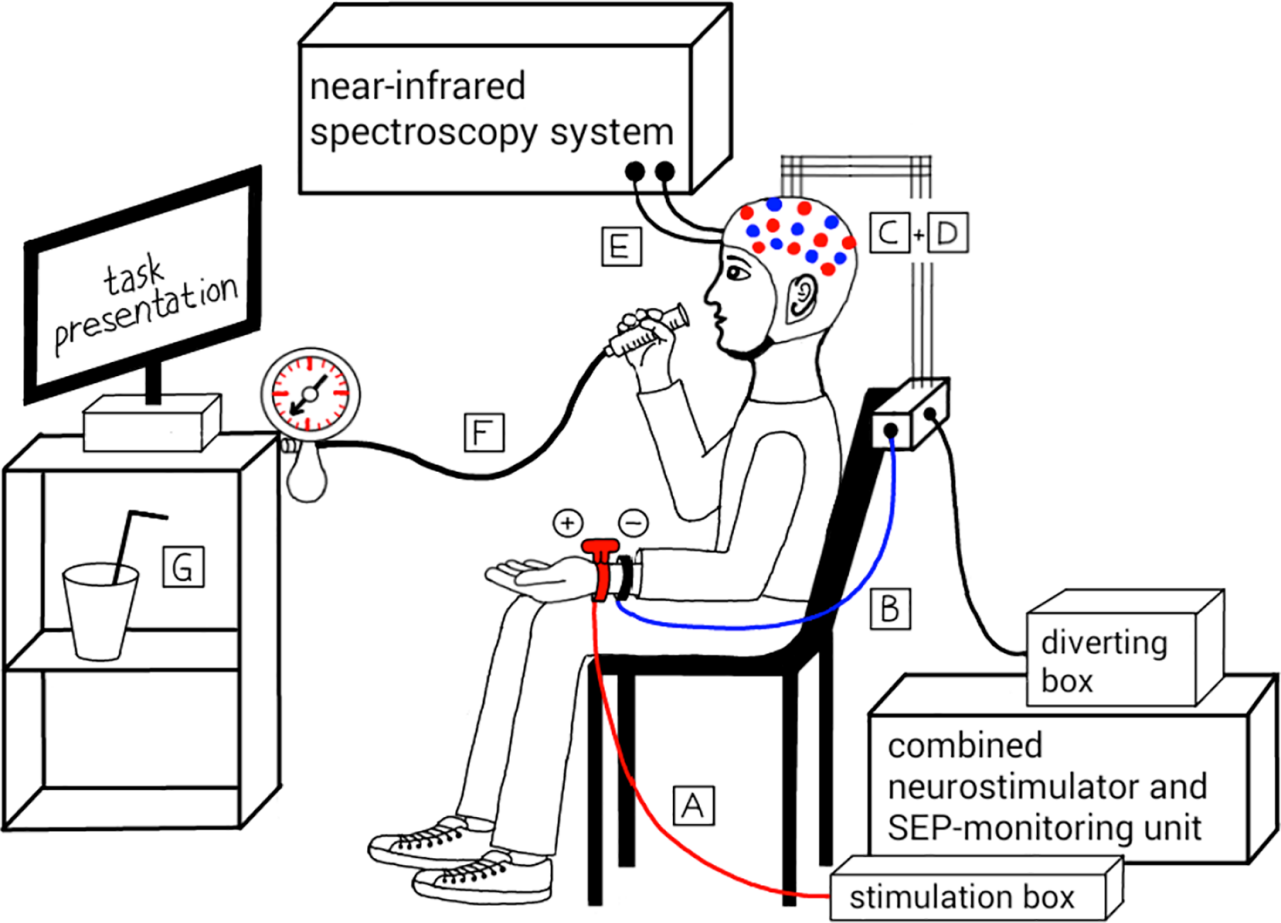
Experimental setup. A blood pressure cuff was connected to a 10 ml plastic syringe. By blowing into the syringe the test person‐produced end‐expiratory pressures of 0 mmHg, 10–20 mmHg, or 30–40 mmHg, depending on the current breathing task. Each task was indicated on a computer screen in front of the test person. MNS was applied manually by the experimental examiner when required during protocol (here shown for left hand MNS). **A**: stimulation electrode, **B**: ground electrode, **C**: diverting electrodes C3′ (right hand MNS) and C4′ (left hand MNS), **D**: reference electrode, **E**: optode bundle, **F**: Valsalva device, **G**: water available during resting periods

Measurements of hemoglobin (Hb) changes were performed with an optical topography system (ETG‐4000, Hitachi Medical Corporation, Tokyo Japan) using continuous‐wave fNIRS (Kamran, Mannan, & Jeong, 2016). A total of 30 optodes (16 emitters alternating with 14 detectors) was attached to an elastic head cap and arranged as two 3 × 5 grids with an area of 14 × 19 cm each covering the left and right hemisphere's SI (Figures 1 and 2). The optode grid was adjusted so that the EEG electrodes for SEP measurement always stood in relation to the same optodes (C3′ between optode 16 and 17, C4′ between optode 27 and 27, Figure 1). With this positioning, which was based on previous studies' findings examining the relation between the international 10–20 system and underlying cortical areas, the SI (postcentral gyrus) was covered (Okamoto et al., 2004; Sato et al., 2006; Steinmetz, Fürst, & Meyer, 1989; Towle et al., 1993). From these 2 × 15 optodes 2 × 22 detector channels were generated, located halfway between emitter and detector. The Hb absorption coefficient is most sensitive to measuring the Hb concentration in spectral bands centered at 700 and 960 nm (Strojnik & Paez, 2013). The applied ETG‐4000 system, therefore, measured absorption changes at the omitted light at 695 nm (dominated by deoxy‐Hb) and at 830 nm (dominated by oxy‐Hb). The inter‐optode distance was 30 mm. The sampling frequency was 10 Hz. Changes of oxy‐Hb and deoxy‐Hb were calculated by a built‐in software using a modified Lambert Beer algorithm (Cope & Delpy, 1988; Kamran et al., 2016). To assess cerebral blood volume (CBV) changes total hemoglobin concentration (total‐Hb) changes were calculated (total‐Hb = deoxy‐Hb + oxy‐Hb).

### 2.4 MNS

Participants were instructed to hold the stimulated hand still in their lap during measurement and to move as little as possible (especially avoidance of jaw and head movement) to avert noise from muscular activity. SI was activated by contralateral MNS at the wrist via two stimulation electrodes (anode and cathode) with the applied ISIS IOM System (Figure 2). Stimulation trains with rectangular pulses (sampling rate 10,000 Hz, time resolution = 0.1 ms, 800 measuring points per 20 s, amplification 100, 30 Hz HP‐filter, notch filter from 5 to 600 Hz) were applied for 20 s with a stimulus frequency of 4.3 Hz. The motor threshold (mean 10.8 mA, range 7–25 mA) was individually identified by visible activation of the Musculus abductor pollicis brevis and then increased for another 3–4 mA to achieve a reliable above‐threshold stimulation (Buchner et al., 2014). A minimal resting interval of 40 s was maintained between two successive stimulation trains. Before starting the experiment, a test run of MNS including a reproduction with 200 summations each was performed (Vogel, 2006). If the test trials produced noisy curves, minimal modifications of the location of the stimulation electrodes and/or EEG electrodes were made in order to optimize both, MNS and SEP recording. Minimal N20 amplitude of 1.0 μV was ensured.

### 2.5 Breathing tasks

Previous studies have shown that breathing against resistance temporarily increases ICP (Brimioulle, Moraine, Norrenberg, & Kahn, 1997; Knauth et al., 2017; Prabhakar, Bithal, Suri, Rath, & Dash, 2007). Four different breathing maneuvers were performed by each participant to modify ICP during the experiment: (a) normal breathing (NB), (b) breath holding for 20 s with 0 mmHg forced expiratory pressure (BH), (c) weak Valsalva maneuver (VM) with 15 mmHg forced expiratory pressure against resistance (V15), and (d) strong VM with 35 mmHg forced expiratory pressure against resistance (V35). To control VMs' intensity, the forced expiratory pressure was measured with a self‐constructed device (10 ml plastic syringe connected to a blood pressure cuff manometer [Pstras, Thomaseth, Waniewski, Balzani, & Bellavere, 2016]). The subject was asked to take a deep breath, and then blow into the syringe maintaining the target pressure for the task's duration. A range for the two pressure levels (10–20 mmHg = V15, 30–40 mmHg = V35) was tolerated since maintenance of one exact pressure level is difficult to achieve for most people. During resting periods, subjects were allowed to breathe freely. For BH subjects were asked to inhale before and to keep their airways open during the breath‐hold, in order to not build up a higher expiratory pressure. For all breathing maneuvers task compliance was monitored by the experimental examiner. Before starting the experiment, a training session was inserted to familiarize participants with different breathing tasks.

### 2.6 Experimental protocol

**Figure 3:**
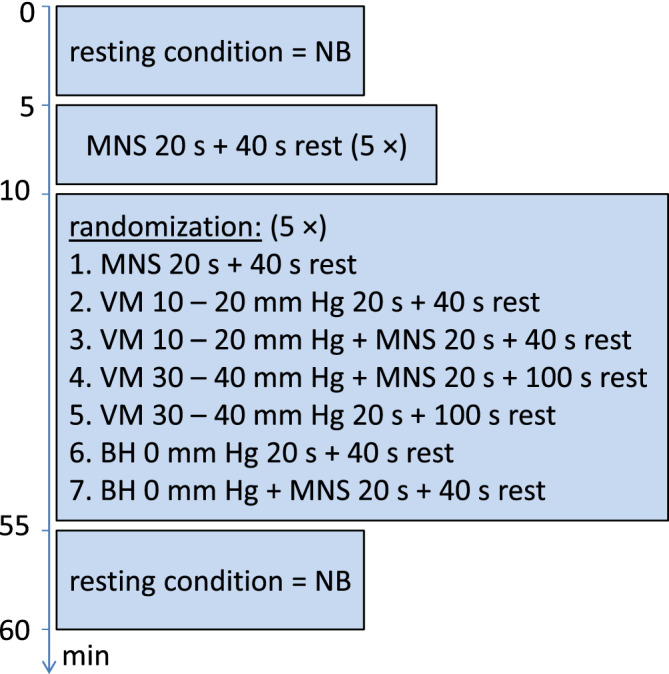
Experimental protocol. The main part comprised five blocks with seven tasks each in random order. NB, normal breathing, MNS, median nerve stimulation, VM, Valsalva maneuver, BH, breath holding

fNIRS measurement consisted of eight different experimental conditions, applied in a Latin square design: rest = NB, MNS + NB, BH, MNS + BH, V15, MNS + V15, V35, and MNS + V35 (Figure 2). All conditions lasted 20 s, with the exception of NB (5 min). Each task was followed by a resting period of at least 40 s. For V35 and V35 + MNS, a longer minimal resting period of 100 s was granted before continuing. Instructions on which task to perform next were shown on a computer screen in front of the subject (Figure 2) via Presentation® software (Version 17.2, Neurobehavioral Systems, Inc. [NBS], Berkeley). After at least 40/100 s, respectively when the subject appeared ready, the examiner initiated the next task via the computer keyboard to proceed the experiment. Depending on the volunteers' different levels of respiratory fitness, sometimes an additional resting time (5–15 s) was granted. Each task was preceded by a 2 s preparation time (visual cue on the screen). An acoustic signal indicated the next task's beginning and a visual cue on the screen was displayed throughout the task's duration. After 20 s the task was ended by another acoustic signal. The examiner was present all along the experiment to give further instructions, if necessary. The measurement took place in a quiet separated room with little external stimuli. Subjects could sip water through a straw during resting times. The fNIRS measurement started with a 5‐min rest during which the participants kept their eyes closed. Then, subjects opened their eyes and five trains of 20 s MNS with an alternating resting time of 40 s were applied. Afterwards, five blocks, containing all experimental conditions in randomized order following a latin square design, were applied (Figure 3). The measurement closed with another 5‐min rest with eyes closed. MNS was performed on the right hand (stimulation of left SI) and, if subjects agreed, the experiment was repeated with left hand MNS (stimulation of right SI) on a different day.

### 2.7 Partial pressure of end‐tidal carbon dioxide after breathing maneuvers

In order to assess the contribution of hypercapnia to the observed changes, additional experiments with Partial pressure of end‐tidal carbon dioxide (PetCO_**2**_) measurements were performed. For this purpose, we employed a calibrated Evita XL Respiratory Ventilator from our intensive care unit (Drägerwerk Co.KGaA, Moislinger Allee 53–55, Lübeck, Germany). On five additional subjects (three women; age range 23–46 years) the same experimental protocol was applied with randomized breathing maneuvers analogue to fNIRS experiments (see Figure 3) except for MNS and fNIRS measurements. The ventilator was adjusted to a nonassisting, continuous positive airway pressure mode with no pressure built up (21% O_2_, 0 mmHg positive end‐expiratory pressure). Subjects inhaled from and exhaled into the ventilating system all through the experiments, except during the maneuvers involving breath holding (BH, V15, and V35). During VMs forced expiratory pressure was built up by blowing into the syringe connected to the manometer described above. To prevent mingling of PetCO_2_ and environmental CO_2_, the mouthpiece of the ventilating system was occluded during breath holding. Analogue to the experimental protocol described above, the subjects performed NB, BH, V15, and V35 10 × each. PetCO_2_ was written down by the examiner during the 2 s preparation time before and after each task. Due to the ventilating system's reaction time to PetCO_2_ changes, values after breathing maneuvers were averaged from the two breaths after the ending of each task.

### 2.8 Data analysis and statistical testing

Data were analyzed with custom‐written scripts using MATLAB® R2018a (The MathWorks, Inc.). Primarily, the data sets were analyzed for each subject individually. Later, a group analysis of all collected data sets was performed. Since the goal of our study was to investigate changes during eICP with a high signal‐to‐noise ratio (SNR), only subject data with a relevant and robust NVC response during NB were included in further analysis. Active measuring channels were selected for each participant by displaying the oxy‐Hb's, deoxy‐Hb's, and total‐Hb's mean time course during NB + MNS (in total 10 × 20 s per subject) for all 22 channels. If NB + MNS showed a sufficient hemodynamic response (oxy‐Hb increase, deoxy‐Hb decrease, total‐Hb increase [Obrig, 2002], by at least 1.5 *SD*), the channel was included into further analysis. In case a subject showed more than one relevant channel, all time‐course data of the active channels were averaged before further analysis. Following a first measuring session with right hand MNS, 44 of the 75 data sets had a sufficiently robust NVC response. Thirty‐eight of the 75 volunteers agreed to repeat measurements on a different day with left hand MNS. From these experiments, 20 showed relevant NVC responses. Thus, overall, 49 experiments (31 right hand MNS, 18 left hand MNS) were excluded from further analysis whereas 64 experiments (44 × right hand MNS, 20 × left hand MNS) with measurable and robust NVC responses and signals from 143 channels (106 left hemisphere, 37 right hemispheres) were included into further analysis. The high dropout rate is discussed in Section 4.2.

#### 2.8.1 Hemoglobin concentration‐time courses in SI

After subtracting baseline values from 11 to 0 s before the beginning of each condition, concentration‐time courses of oxy‐Hb, deoxy‐Hb, and total‐Hb were first averaged for each of the seven tasks across all trials within each subject and then plotted as mean with a ± 95% confidence interval across all 44 subjects. To control for systematic data trends, corresponding concentration changes during four consecutive time intervals of 66 s were taken from the resting period conditions and averaged. It is known that VMs induce large global oxygenation changes (Wu, Bandettini, Harper, & Handwerker, 2015). Therefore, an additional subtraction analysis was performed to extract the functional oxygenation signal from recorded global changes. In detail, within each subject, the averaged Hb time courses for breathing tasks without MNS were linearly subtracted from corresponding averaged Hb time courses for breathing tasks with simultaneous MNS.

#### 2.8.2 SEP‐analysis

SEPs were averaged for each condition across all trials within each subject and then averaged across all 44 displayed data sets. In order to account for SEP differences due to tissue resistance (skin and skull), normalization was performed before averaging across subjects. Averaged within‐subject SEP time courses were divided by the subject's maximum SEP amplitude. Following the experimental protocol, every data set aimed at averaging across 3 × 5 SEPs (BH, V15, and V35) and 1 × 10 SEPs (NB) for the respective condition. In 12 of 64 experiments, SEPs from individual stimulation trains (17 out of the totally planned 1,600 trains across all 64 experiments) could not be saved due to a technical failure and were therefore interpolated from the remaining obtained SEPs of the same condition when averaging.

#### 2.8.3 Statistical analysis of time‐course data

To statistically assess Valsalva‐induced eICP's influence on functional responses to MNS, the resulting mean amplitude of deoxy‐Hb, oxy‐Hb, and total‐Hb during MNS (9–20 s after initiation of 20 s right hand MNS) and the mean SEP amplitude were calculated for each condition. A paired *t* test (including Bonferroni correction) was applied for testing differences between baseline conditions and breathing tasks. Results with *p* < .05 were considered statistically significant, results with *p* < .0005 were considered highly significant.

#### 2.8.4 Functional imaging data

While the analysis explained in Section 2.8.3 is suitable to assess the breathing maneuvers' effect on the cortical Hb time courses during MNS in a predefined region of interest, it does not consider the image contrast induced by vascular changes evoked by MNS. To this end, we carried out an additional analysis examining the breathing maneuvers' influence on the feasibility of performing functional brain imaging based on oxy‐Hb and deoxy‐Hb. A subgroup of 16 subjects was selected, showing typical hemodynamic responses during MNS for the left and right SI during NB. The oxy‐Hb and deoxy‐Hb time courses measured by the 22 channels were linearly interpolated to a 5 × 9 image matrix for each hemisphere (90 pixels in total). A pooled *t* test across all subjects was conducted on each pixel comparing the mean amplitude of oxy‐Hb and deoxy‐Hb during the 20 s of MNS to the mean amplitude of the baseline at −7 to −2 s before the onset of MNS. In order to account for the global changes evoked by the different breathing maneuvers, time course data from BH, V15, and V35 without MNS were subtracted from the corresponding time courses with MNS across all subjects to conduct the previously mentioned pooled *t* test. The results were color‐coded and displayed as functional maps.

## 3 RESULTS

**Figure 4:**
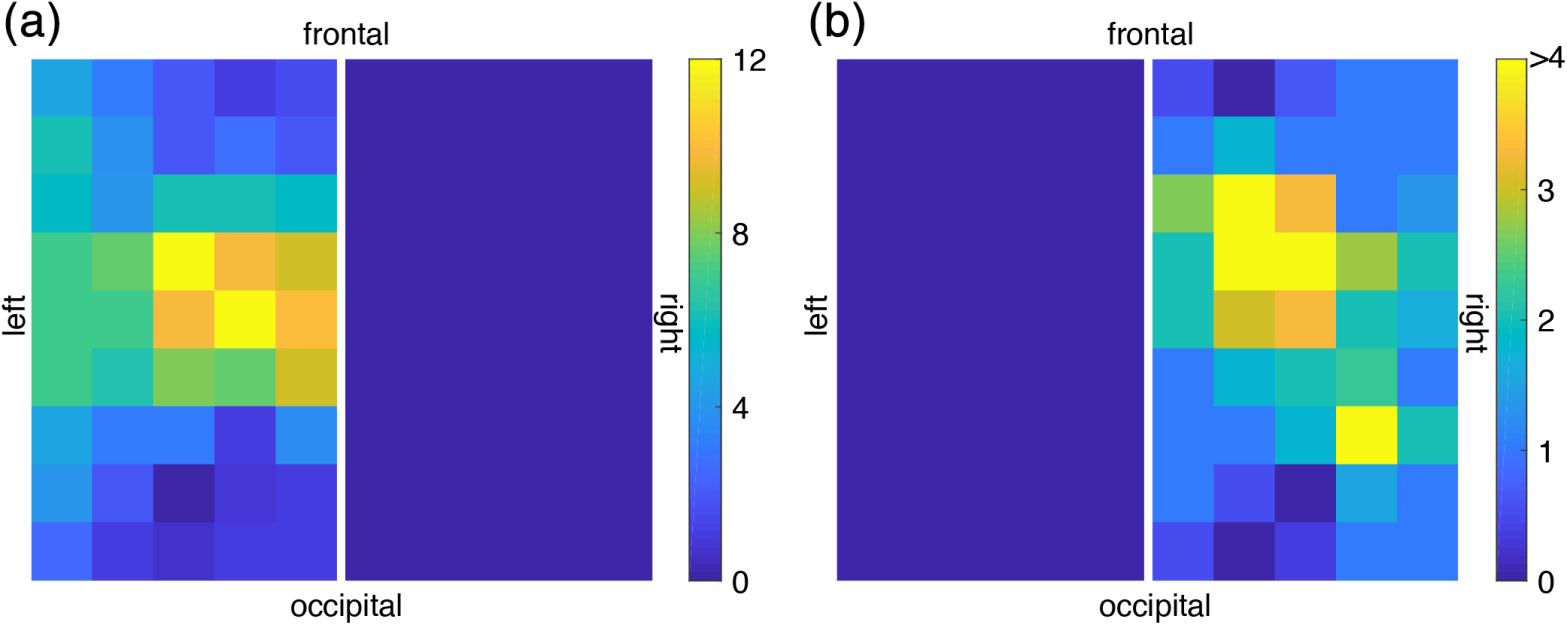
Localization of activated channels with regard to the optode grid (interpolated to a 5 × 9 array for each hemisphere) during MNS applied to (a) right hand (*n* = 44), (b) left hand (*n* = 20). The colored scale indicates the total number of activated measuring channels. Due to a lower number of subjects in (b) compared to (a) the scale shows the same color for areas with ≥4 activated channels to increase the image contrast

Sixty‐four experiments (44 × right hand MNS; 20 × left hand MNS) from 48 subjects (16 of 44 participants for right hand MNS also participated in left hand MNS; 4 participants were only analyzed for left hand MNS) with distinguishable SI activation and NVC responses and data from 143 channels in total (106 left hemisphere; 37 right hemisphere) were included in the time‐course analysis. In 16 subjects, the experiment was carried out for both sides (right and left hand MNS) and delivered discernible hemodynamic SI responses contralaterally for both sides. Figure 4 illustrates the number of channels with functional hemoglobin changes during right (a) and left (b) hand MNS. The additional data from left hand MNS were used to assess functional imaging but were not included in the time course analysis to avoid a statistical bias since these data were not obtained from independent samples.

### 3.1 Cortical hemoglobin concentration time courses during MNS and eICP

**Figure 5:**
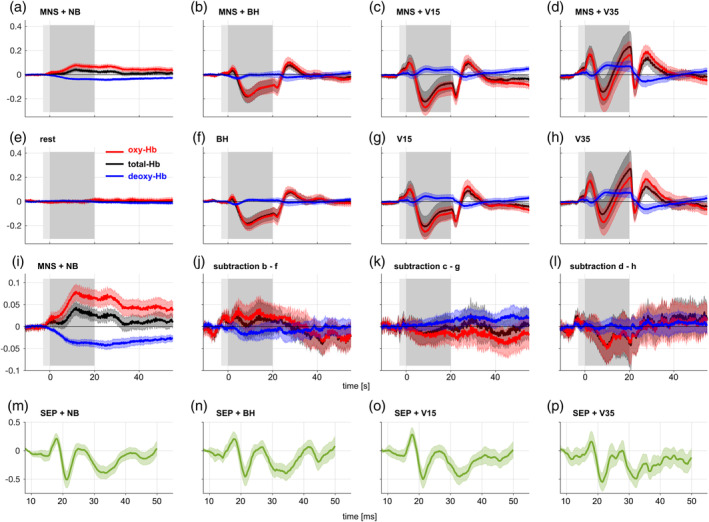
Influence of eICP on vascular responses (time courses of regional oxy‐, deoxy‐, and total‐Hb) and neuronal activity in the SI during MNS. Displayed are time courses averaged across all trials of the respective condition within each subject, then depicted as mean across subjects with a ± 95% confidence interval from 44 left SI areas (right hand MNS) from 44 subjects. Light gray box: preparation time (2 s) depicted on a computer screen, dark gray box: time interval per condition (20 s). (a) During MNS, a typical local hyperoxygenation (increase in oxy‐ and total‐Hb, decrease in deoxy‐Hb) becomes apparent in contralateral SI. When accompanied by the breathing maneuvers BH, V15, and V35 (b–d), this vascular response cannot be discerned. Instead, a large global multiphasic response known to be associated with the applied breathing maneuvers is seen. Corresponding vascular responses without MNS (f–h) follow a very similar time course. (i) contains the same data as (a) but on a larger scale. When subtracting (f–h) from (b–d) the MNS evoked component of the vascular response should become apparent (j–l). However, although the oxy‐Hb time course during MNS + BH suggests a trend toward a small hyperoxygenation, no significant MNS induced local hyperoxygenation can be recovered. At the same time, neuronal activity as measured by SEPs remains largely unchanged during the different breathing maneuvers (m–p). This suggests that oxy‐Hb and deoxy‐Hb no longer signalize neuronal activity in the SI during eICP

Time courses of regional deoxy‐Hb, oxy‐Hb, and total‐Hb changes during the different experimental conditions averaged across subjects are shown in Figure 5. During MNS induced SI activation and NB (5 × 20 s before randomization and 5 × 20 s during randomization), a typical NVC response consisting of oxy‐Hb and total‐Hb increase and simultaneous deoxy‐Hb decrease is depicted (Figure 5a). Oxy‐Hb values increase to a maximum of 0.08 mM*mm and total‐Hb values to a maximum of 0.04 mM*mm 11.8 s after MNS onset, slowly approaching baseline values after stimulus cessation. Deoxy‐Hb starts descending with MNS onset and reaches a plateau of −0.037 mM*mm 8.5 s after MNS onset. After stimulus cessation, deoxy‐Hb slowly recovers toward baseline values. The concurrent neuronal activity measured by EEG shows a typical SEP (5 m). Figure 5b–d shows the Hb concentration time courses during MNS and accompanying escalating breathing maneuvers to achieve eICP. During all breathing tasks, the typical NVC response is not discernable. Instead, a known large polyphasic oxy‐Hb and total‐Hb change can be observed. A first positive peak becomes apparent 2 s after the onset of the breathing maneuver, which increases in amplitude with escalation of the breathing maneuver (0.055 mM*mm during BH, 0.1 mM*mm during V15, 0.17 mM*mm during V35). At 8 s after the onset of the breathing maneuver, a first minimum occurs (−0.18 mM*mm after BH). This minimum shows a trend toward a larger amplitude during V15 (−0.27 mM*mm) and a smaller amplitude during V35 (−0.2 mM*mm). When applying V15 and V35, it is followed by a second maximum immediately after the end of the breathing maneuver which is of negative amplitude after V15 (−0.1 mM*mm) but reaches positive values during V35 (0.2 mM*mm). A second minimum 2 s after the end of the breathing maneuver is seen after V15 (−0.18 mM*mm) and has a smaller amplitude after V35 (−0.11 mM*mm). Finally, before recovery to baseline values, a third maximum is reached 8 s after the end of the breathing maneuver that increases in amplitude with escalating breathing maneuvers (0.09 mM*mm during BH, 0.1 mM*mm during V15, 0.15 mM*mm during V35). When considering deoxy‐Hb, a different performance is observed with escalating breathing maneuvers. While BH does not lead to relevant changes in deoxy‐Hb, V15, and V35 are accompanied by a sluggish increase in deoxy‐Hb reaching a temporary plateau (0.05 mM*mm during V15, 0.08 mM*mm during V35) and recovering to baseline values 4 s after the end of the breathing maneuver. Visually comparing these Hb changes during BH, V15 and V35 and accompanying MNS (Figure 5b–d) with the corresponding condition without MNS (Figure 5f‐h) a relevant difference is not suggested. When doing a subject to subject analysis by subtracting the time courses without MNS from the corresponding time courses with MNS (Figure 5j‐l) this impression is confirmed. Apart from a small non‐significant trend toward an increase in oxy‐Hb and total‐Hb during BH + MNS, no NVC response can be recovered. Instead, no MNS specific changes in oxy‐Hb, deoxy‐Hb, and total‐Hb can be distinguished. However, neuronal activity evoked by MNS is preserved as SEPs concurrently recorded remain largely unchanged (Figure 5n–p). This suggests that Hb concentration changes during neuronal activation can be attenuated almost completely by breathing maneuvers with eICP.

### 3.2 Statistical analysis of time course data

**Figure 6:**
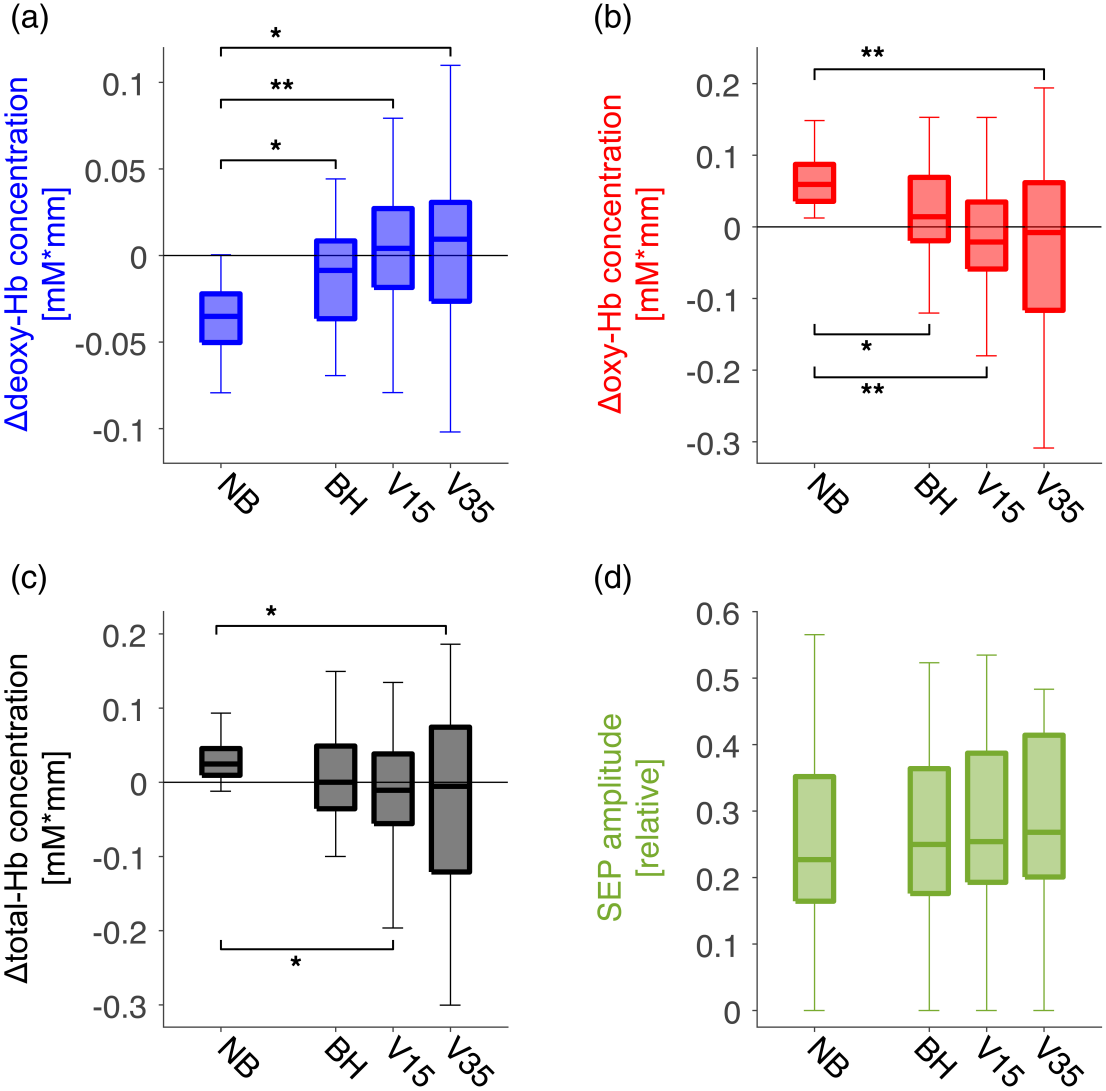
Box plots across experiments (44 SI areas from 44 subjects) with mean amplitudes of functional responses during MNS. During NB, deoxy‐Hb (a), oxy‐Hb (b) and total‐Hb (c) show MNS‐evoked concentration changes corresponding to a typical hyperoxygenating NVC response. With all three escalating breathing maneuvers (NB, V15, and V35), this response is abolished with significant alterations of the concentration change during MNS (*p < .05, **p < .0005, Paired *t* test with Bonferroni correction). (d) shows relative SEP amplitudes (absolute values) during NB and during the different breathing maneuvers with no apparent change signifying preserved neuronal activity during eICP

To further assess the NVC response during eICP, the mean amplitude of deoxy‐Hb, oxy‐Hb and total‐Hb at the maximum response (9–20s after MNS onset) as well as the mean SEP amplitude (absolute values, that is, positive and negative values) of each breathing condition were analyzed across subjects (*n* = 44). For BH, V15, and V35 the concentration time courses without MNS were subtracted from the corresponding concentration time courses with MNS (as described for Figure 5i–l). The resulting data are presented as box plots in Figure 6. All three escalating breathing maneuvers (BH, V15, and V35) significantly alter the MNS evoked changes in both, deoxy‐Hb (Figure 6a) and oxy‐Hb (Figure 6b). During NB, oxy‐Hb and total‐Hb increase while deoxy‐Hb decreases which reflects a typical NVC response. However, this functional change can no longer be observed during the applied breathing maneuvers. Concurrently measured SEP amplitudes (Figure 6d) do not show a relevant change. Taken together, this reflects a significant attenuation of deoxy‐Hb and oxy‐Hb changes during MNS‐induced neuronal activation caused by breathing maneuvers with eICP. Table 1 contains the results of the corresponding statistical tests.

**Table 1:** Paired *t* test (visualized in Figure 6)

Paired *t* test	*p*‐value	*t*‐value
**Deoxy‐Hb** a		
NB vs. BH	0.033548 *	27.634
NB vs. V15	0.000011 2 **	53.967
NB vs. V35	0.000482 *	42.285
BH vs. V15	0.195124	2.028
**Oxy‐Hb** b		
NB vs. BH	0.001202 *	−39.334
NB vs. V15	0.000004 **	−56.833
NB vs. V35	0.000030 **	−50.947
BH vs. V15	0.190965	−20.379
**CBV** c		
NB vs. BH	0.553363	−15.101
NB vs. V15	0.023906 *	−28.924
NB vs. V35	0.019387 *	−29.707
BH vs. V15	1	−0.93243

a Deoxygenated hemoglobin concentration,

b Oxygenated hemoglobin concentration,

c Cerebral blood volume ≙ total hemoglobin concentration. *p*‐values were determined and Bonferroni corrected (primary *p*‐values multiplied by four) for 12 tests concerning relative changes in deoxy‐Hb, oxy‐Hb, and total‐Hb, of which 5 were significant (*p < .05) and 3 were very significant (**p < .0005). For deoxy‐Hb all applied tests (4) except for BH versus V15 were significant or very significant, for oxy‐Hb three out of four tests were and for total‐Hb two of the applied tests were statistically significant. In detail, the differences between deoxy‐Hb and oxy‐Hb amplitudes for NB conditions and conditions with eICP (BH, V15, and V35) were all significant. The total‐Hb amplitude showed a significant difference between NB and V15 as well as between NB and V35. This underlines the time course analysis' finding that functional deoxy‐Hb amplitudes are significantly smaller during V15 and V35 when compared to NB and BH. The oxy‐Hb's and total Hb's mean amplitude also gets smaller with the escalation of breathing tasks. These findings support the theory of a deoxy‐Hb decoupling mechanism with an increasing positive difference and the passing of the zero line during the breathing maneuvers' progression.

### 3.3 Functional imaging data

**Figure 7:**
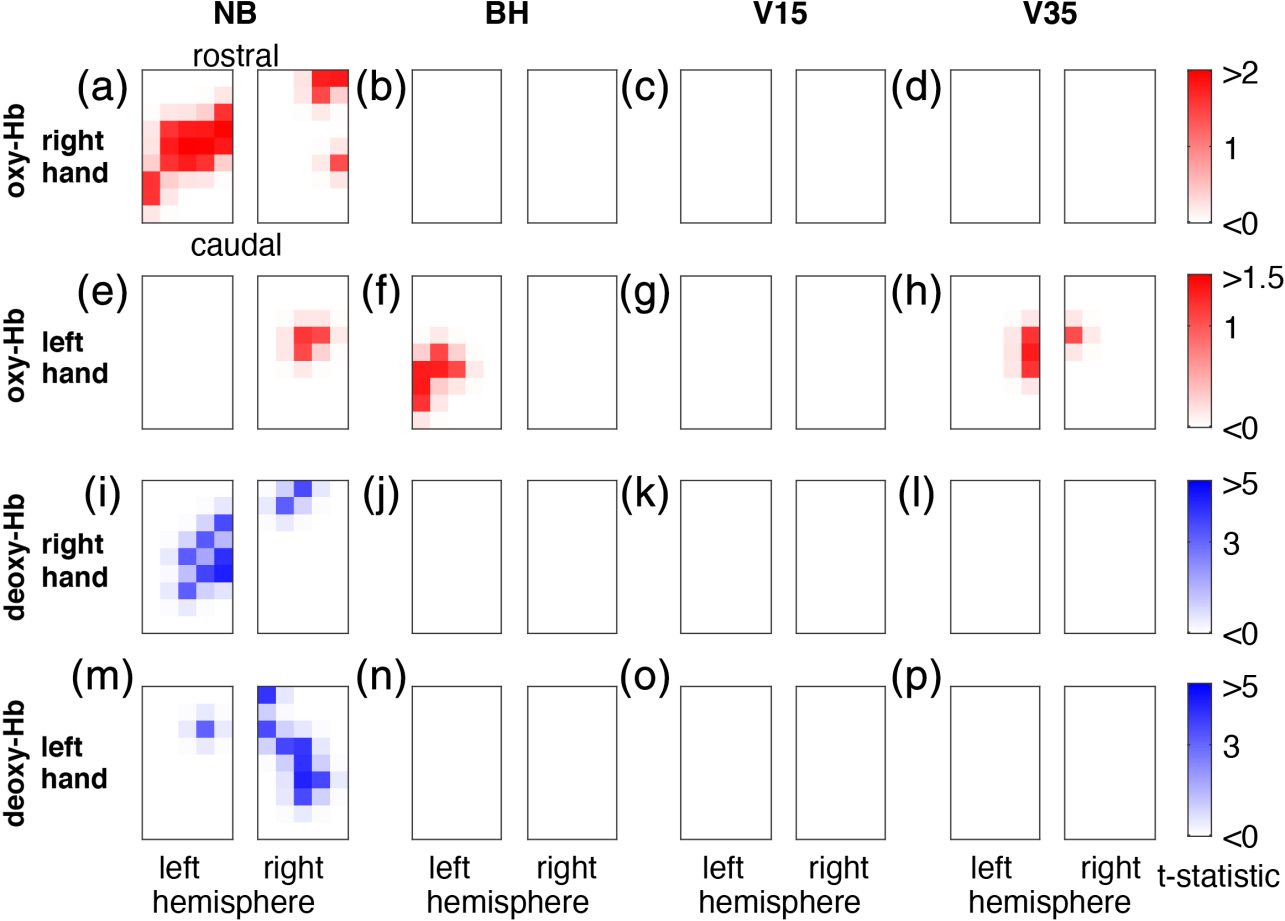
Effect of ICP elevation via breath holding tasks on fNIRS imaging. Interpolated from the bilateral 3 × 5 optode grid an interpolated bilateral 5 × 9 image matrix with 45 pixels was created for each hemisphere and every condition. Each pixel was subjected to a pooled *t* test comparing its mean amplitude during MNS (0–20s) with its mean amplitude before MNS (−7 to −2 s before MNS initiation) in all subjects (*n* = 16). Top and second row: oxy‐Hb based functional maps evoked by right hand MNS (a‐d) and left hand MNS (e–h), third and bottom row: deoxy‐Hb based functional maps evoked by right hand MNS (i–l) and left hand MNS (m–p). During NB, both oxy‐Hb and deoxy‐Hb based functional imaging robustly identifies the activated contralateral SI (a, e, i, m). However, with parallel breathing maneuvers, no activation can be localized. Functional imaging based on Hb does not identify the activated SI when accompanied by VM‐induced ICP elevation

Functional neuroimaging displays visible contrasts between areas that differ in their functional state and thus brain maps of activated areas can be obtained. To assess Valsalva‐induced eICP's influence on functional neuroimaging based on oxy‐Hb and deoxy‐Hb, an additional imaging analysis was performed in all subjects in which distinct functional oxygenation changes were obtained during right and left hand MNS (*n* = 16). In order to obtain a complete image rather than irregularly positioned points, all individual measuring channels were converted to an image matrix before further analysis. To this end, the fNIRS data of each hemisphere's grid (2 × 15 channels) were linearly interpolated, resulting in an image matrix of 5 × 9 pixels for each hemisphere. In each resulting pixel, a pooled *t* test was performed comparing its mean amplitude during MNS (0–20 s) with its mean amplitude before MNS initiation (−7 to −2 s before MNS) in all subjects. For the BH, V15, and V35 paradigms, the above‐mentioned subtraction analysis was performed before this test in order to remove global changes associated with the breathing maneuvers. Figure 7 displays the results of this functional imaging analysis. During NB, both oxy‐Hb and deoxy‐Hb based functional imaging clearly identifies the activated SI (Figure 7a,e,i,m). However, in none of the conditions involving breathing maneuvers, this brain mapping can be reproduced. Corroborating the time course data, this analysis shows that functional imaging based on oxy‐Hb and deoxy‐Hb concentration time courses is not capable to localize neuronal activation during Valsalva‐induced eICP.

### 3.4 PetCO_2_ after breathing maneuvers

In order to assess the contribution of hypercapnia to the observed changes, additional experiments with PetCO_**2**_ measurements applying the same experimental protocol (except for MNS and fNIRS measurements) were performed. Figure Sa, contains single subject plots averaged across blocks with equal breathing maneuvers of PetCO_**2**_ measured before and after the particular breathing maneuver and before the adjacent breathing maneuver. While an expected variance of baseline PetCO_**2**_ measurements is visible, subjects generally show an increase after BH, V15, and V35 with return to baseline values before the next maneuver. Figure Sb displays box plots of absolute changes (top) as well as relative percentage of changes (bottom) after each breathing maneuver and before the adjacent maneuver. At the end of NB, the median absolute change in PetCO_**2**_ was 0.3 mmHg (95% CI 0.0–0.5) whereas PetCO_**2**_ rose by 3.9 mmHg (95% CI 3.1–4.7), 3.7 mmHg (95% CI 3.0–4.4) and 3.4 mmHg (95% CI 2.3–4.5) after BH, V15, and V35 maneuvers respectively (Figure Sb, top). These changes translate into percent changes relative to PetCO_**2**_ before the breathing maneuvers of 0.8% (95% CI 0.1–1.5), 8.9% (95% CI 5.9–12.0), 9.4% (95% CI 7.0–11.8), and 10.4% (95% CI 6.1–14.6) for NB, BH, V15, and V35 maneuvers, respectively. Before the next breathing task, median values were 0.1 mmHg (95% CI −0.2–0.3), 0.6 mmHg (95% CI 0.0–1.2), −0.2 mmHg (95% CI −0.5–0.1) and − 0.9 mmHg (95% CI −1.1 to −0.7) for NB, BH, V15, and V35 maneuvers, respectively. These changes correspond to relative changes of 0.5% (95% CI −0.3–1.2), 1.9% (95% CI 0.4–3.3), −0.3% (95% CI −1.1–0.5) and − 2.2% (95% CI −2.8 to −1.6) and indicate a return to baseline values after each task.

## 4 DISCUSSION

Employing fNIRS, our study investigated the influence of eICP induced by breathing maneuvers on oxy‐Hb and deoxy‐Hb during NVC in the activated SI. Our main findings are:

1. During NB, MNS produces a typical rise of Hb concentration in the contralateral SI resulting in hyperoxygenation, that is, increase in oxy‐Hb and decrease in deoxy‐Hb. EEG recording reveals typical SEPs as direct correlate of neuronal activity.
2. During the different breathing maneuvers with eICP, a large global polyphasic oxygenation response occurs in SI, increasing in amplitude with escalating maneuvers from BH to V15 and V35.
3. When subtracting oxy‐Hb and deoxy‐Hb time courses during escalating breathing maneuvers without MNS from corresponding time courses with MNS, the functional hyperoxygenation response is diminished to a slight trend (BH) or vanishes altogether (V15 and V35). As parallel SEPs remain largely unchanged, this suggests that eICP attenuates oxy‐Hb and deoxy‐Hb concentration changes during neuronal activation in the SI.
4. Functional imaging with fNIRS correctly localizes the corresponding SI during MNS + NB but fails to do so during breathing maneuver induced eICP. Therefore, functional imaging of the SI based on deoxy‐Hb is impaired during eICP.
  In the following, we will address methodological issues of our study and discuss possible implications.

### 4.1 Breathing maneuvers as means of ICP elevation

Experimental invasive ICP elevation by mock CSF infusion has been shown to reduce and even reverse the functional deoxy‐Hb response in rat SI (Füchtemeier et al., 2010). Translated to patients undergoing brain mapping with BOLD‐fMRI this has fundamental consequences as activated areas might not be localized correctly in the possibly damaged brain, for example, in brain tumor patients (Lindauer et al., 2010). When attempting to transfer this effect to and assess it in humans, graded VMs are an obvious noninvasive mean for eICP attainment. Depending on the amplitude of the applied intrathoracic pressure, jugular venous outflow is reduced which causes a displacement of CSF into the cranial cavity (Wostyn, Audenaert, & De Deyn, 2009) and elevates ICP (Williams, 1981). It should be noted that the applied BH maneuver, although formally not a VM, would also lead to a small increase of intrathoracic pressure with a probable small increase in ICP (Thomason & Glover, 2008; Wu et al., 2015).

Since breath holding and VMs increase alveolar CO_2_ concentration, the effects of relative arterial hypercapnia might interfere or contribute to the observed changes. For this reason, we performed additional PetCO_2_ measurements confirming a PetCO_2_ increase by about 10% or 4 mmHg evoked by breath holding maneuvers. This raises the question to what extent the effects observed in our study can be attributed to an arterial PetCO_2_ increase. With a rise of PetCO_2_ by 6 mmHg, a reduction of functional CBF increases by about 30% can be expected (Whittaker, Driver, Bright, & Murphy, 2016). Considering the obtained fNIRS responses to MNS (Figure 5i), a reduction of this magnitude would still be detectable in our data analysis. Thus, the observed changes exceed the magnitude expected from a PetCO_2_ increase by 4 mmHg. In addition, the temporal profile of our fNIRS responses to the breathing maneuvers argues against a sole CO_2_ effect. On the one hand, the gradual return of oxy‐Hb and deoxy‐Hb to baseline values after the breathing maneuvers observed in our experiments could suggest a gradual washout of accumulated arterial pCO_2_. On the other hand, the complex multiphasic response observed during the maneuver has a prompt onset too fast to be attributed to blood gas changes. Furthermore, an accumulation of arterial pCO_2_ over the course of the experiment could be put forward as a relevant confounder of our data. However, this seems unlikely, considering the return of elevated PetCO_2_ to baseline values after the next breathing maneuver in our experimental data. Therefore, while breath holding leads to hypercapnia (Lindholm & Lundgren, 2006; Sasse, Berry, Nguyen, Light, & Mahutte, 1996; Seddon, Thacker, Jurd, & Loveman, 2014), eICP effects probably outweigh hypercapnia effects. However, future fNIRS and fMRI studies should include PetCO2 monitoring, as it can change not only by forced breathing maneuvers: a speech task‐induced mild PetCO_2_ decrease by 4% produced relevant cortical oxygenation changes in a recent fNIRS study that could be falsely attributed to neuronal activation (Scholkmann, Gerber, Wolf, & Wolf, 2013).

Another confounder of our eICP paradigm stems from its transient characteristic, that is, the 20 s breathing maneuver does not allow for a new equilibrium of total‐Hb, oxy‐Hb, and deoxy‐Hb to take place. However, the advantage of this approach with fast recovery is that different eICP levels can be applied in randomized order, which in turn increases the validity of the observed effect. By subtracting trials without parallel MNS from trials with MNS we were able to examine the effect of breathing maneuvers. Confirming our previous study's findings in the human MI (Knauth et al., 2017) and the initial animal study's results (Füchtemeier et al., 2010) we observed an eICP correlated decrease in the deoxy‐Hb response. Taken together, though the results of this study differ in some ways (discussed below), we are confident that Valsalva‐induced eICP is an appropriate mean to study the effect of eICP on NVC related changes in oxy‐Hb and deoxy‐Hb in human SI.

While the alignment of experimental protocols between our previous study in the human MI (Knauth et al., 2017) and our current study on SI allows for comparability, it also leads to a common limitation. A VM inevitably leads to global extracranial blood flow changes, for example, from the scalp, possibly interfering with fNIRS measurements. This problem is discussed in more detail in our previous study on MI (Knauth et al., 2017). Briefly summarized, while superficial scalp veins have been shown to contribute to fNIRS signals (Kirilina et al., 2012), fNIRS measurements with different penetration depths found a rather small contribution from superficial layers (Tsubaki, Kojima, Furusawa, & Onishi, 2013) during VMs. In addition, a combination of NIRS with Laser Doppler Flowmetry did not reveal a relevant influence of surface vasomotor activity on cortical NIRS data (Aletti et al., 2012). Therefore, although we cannot exclude a small contribution of extracranial oxygenation signals the predominant fNIRS signals from our study are most likely of cortical origin. Nevertheless, the used NIRS setup with only one penetration depth constitutes a limitation of our study. Future studies on systemic changes should be done with optimized NIRS systems which are able to discriminate signals from scalp flow and other signals not related to cortical blood flow.

### 4.2 Dropout rate and SNR

Since we aimed to investigate pathological influences on physiological NVC our experimental setup was dependent on a strong and unambiguous cortical MNS oxygenation response during NB. If such a response could not be detected during baseline conditions with NB, an assessment under nonbaseline conditions (= eICP) would not have rendered value to this endeavor. Therefore, the original data from 113 experiments were confined to 64 which were further analyzed. Forty‐nine sets were excluded because no discernable oxygenation change was observed during MNS + NB after averaging across trials post hoc. The dropout rate was therefore even higher than in our previous study on the human MI where 13 from 59 experimental subjects were excluded (Knauth et al., 2017). Such a preselection based on SNR bears the risk of systematic bias. Our paradigm only included 10 repetitions for MNS + NB which might have been too few for many experimental subjects to achieve a sufficient SNR. In theory, additional averaging by adding more experimental blocks could have increased the number of experiments with an unambiguous and clear oxygenation change during MNS. However, due to the exhausting VM paradigm, a corresponding lengthening of the experimental duration was not a practical option. Instead, we increased the number of experimental subjects. We also considered the alternative of applying an event‐related design instead of a block design to achieve a higher SNR (Klingner et al., 2011). However, we wanted to ensure sufficient comparability to the analogue study on the MI (Knauth et al., 2017). The encountered heterogenous SNR is not unexpected. fNIRS studies have described multiple SNR impairments, with assumed explanations including age, cooperation, anatomical characteristics such as skull thickness, inaccurate placements of NIRS optodes, wavelength dependence of sensitivity and CSF layers, physiological phenomena relating to brain activation and systemic changes in blood pressure or heart rate (Moriguchi et al., 2017; Sato et al., 2005; Strangman, Franceschini, & Boas, 2003). In summary, we are convinced that our confining of the study population to subjects with high SNR does not distort the results of our study but instead raises their validity.

### 4.3 Valsalva‐induced eICP and multiphasic oxy‐Hb and deoxy‐Hb changes in SI

One main goal of this study was to examine whether the effect observed in a previous study employing Valsalva‐induced eICP on NVC in the human MI (Knauth et al., 2017) could be reproduced in the human SI. To this end, the experimental protocols were equalized. This enabled us to compare the effect of Valsalva‐induced eICP on oxy‐Hb and deoxy‐Hb in the SI to the corresponding effect in the MI. The Valsalva‐evoked response profile and magnitude of oxy‐Hb, total‐Hb, and deoxy‐Hb were very similar in the two studies, confirming our interpretation of this response as a global stimulus affecting several cortical areas similarly. Oxy‐Hb and total Hb followed a multiphasic response profile with three peaks and two troughs. Deoxy‐Hb had a transient decrease at onset and end of the breathing maneuver and a plateau in between. The level of this plateau increased with eICP along the three breathing maneuvers. Former studies employing fNIRS (Tsubaki et al., 2013) and BOLD‐fMRI (Henderson et al., 2002) found similar transients during VMs. Considering the multiphasic response, it is likely that not only ICP but also other physiological alterations (systemic blood pressure, arterial pCO_2_, autonomic nervous system changes with vascular compartmental redistribution) are responsible for the oxygenation changes observed during VMs. Thus, while Valsalva leads to an ICP increase it also leads to other systemic changes visible in cortical oxygenation. For a more detailed interpretation of this multiphasic response, we refer to our previous study (Knauth et al., 2017) where we describe a possible sequence of congestion and re‐opening of arteries and veins as a correlate of this oscillation of oxy‐Hb, total‐Hb, and deoxy‐Hb.

### 4.4 eICP and attenuation of functional oxy‐Hb increase and deoxy‐Hb decrease

When subtracting oxy‐Hb and deoxy‐Hb time courses obtained during eICP by escalating breathing maneuvers without corresponding MNS from time courses with MNS we could not recover a reliable functional activation response in SI. During BH, a trend toward a sustained NVC response is visible while, on the one hand, both, V15 and V35, did not differ relevantly between trials with MNS from trials without and, on the other hand, SEPs showed preserved neuronal activation. Once again, eICP, therefore, prevented deoxy‐Hb changes during neuronal activation, raising doubts on the validity of deoxy‐Hb‐based functional brain imaging under eICP conditions. However, different from these results, our previous study on human MI found a dissociation of oxy‐Hb and deoxy‐Hb with eICP (Knauth et al., 2017). While a robust deoxy‐Hb response to finger tapping could be recovered during BH, it was attenuated compared to NB. During V15 and V35 deoxy‐Hb did not show a response. At the same time, oxy‐Hb had a prevailing response to finger tapping during BH, V15, and V35. This phenomenon of an oxy‐Hb versus deoxy‐Hb decoupling did not occur in our current study on the human SI. Nevertheless, we were able to closely reproduce the results of the framing experimental paradigm with very similar oxy‐Hb and deoxy‐Hb time courses during functional activation as well as during the different breathing maneuvers. A neuronal deactivation (e.g., caused by overlying exhausting motor activity during the breathing maneuvers on MNS) could explain this difference but is very unlikely since SEPs were not influenced relevantly by VMs. Neuroanatomical differences could possibly contribute to this finding as MNS activation typically takes place in the more superficial cortical layer IV while finger tapping is dominantly evoked by neuronal activity in cortical layer V (Trepel, 2017). A less robust deoxy‐Hb response during SI activation, when compared to MI activation, is in accordance with the observation that MNS has been used in comparably few fNIRS studies with most of them reporting subjects without discernable functional Hb concentration changes. One fNIRS study found SI activation after MNS in only eight out of 12 subjects (Niederhauser, Rosenbaum, Gore, & Jarquin‐Valdivia, 2008) while another fNIRS study reported a missing response in 3 of 10 subjects and inconsistent data in three of the remaining subjects (Tanosaki et al., 2001). Another study did not report any subjects with failed SI activity but showed data of only medium SNR (Takeuchi et al., 2009). When using fMRI during MNS one study did not obtain functional activity in two of nine subjects, while all nine subjects showed significant activation during a finger opposition task (Spiegel, Tintera, Gawehn, Stoeter, & Treede, 1999). Another fMRI study found MNS activation in the SI to be considerably dependent upon attention, although it is not clear whether this BOLD signal variance was due to differences in neuronal activity since no parallel EEG recording was obtained (Backes, Mess, van Kranen‐Mastenbroek, & Reulen, 2000). Moreover, one study suggests a frequency dependence of the obtained fMRI responses upon MNS (Kampe, Jones, & Auer, 2000). It has been demonstrated that sensory stimuli can evoke EEG responses without concurrent positive BOLD‐fMRI response. On the contrary, a decrease in BOLD contrast was recorded and interpreted as a focal deactivation due to a reduced neuronal baseline activity induced by inhibitory interneurons (Blankenburg et al., 2003). While we were able to recover functional oxygen changes in the motor cortex by subtracting responses to BH without responses with finger tapping (Knauth et al., 2017), only a weak trend toward functional oxygenation changes remains when applying BH to MNS and SI. It is therefore tempting to speculate that breathing differences also contributed to the variance in the previously mentioned studies and the general problem of acquiring robust MNS induced functional Hb responses. Variance might be reduced by training subjects to pace their breathing and—if breath holding is applied—to hold breath after exhalation instead of inspiration (Handwerker, Gazzaley, Inglis, & D'Esposito, 2007).

### 4.5 Functional brain imaging of SI based on oxy‐Hb and deoxy‐Hb changes

The analysis of eICP induced changes on oxy‐Hb and deoxy‐Hb during MNS was accomplished with two different methods. In one approach, averaged time course data of activated fNIRS channels were analyzed with statistical tests on amplitude changes as described and discussed above. In the second approach, we assessed functional imaging data obtained from a subgroup of 16 subjects in which MNS‐induced changes of oxy‐Hb and deoxy‐Hb were obtained for both left‐ and right‐side stimulation. By performing this analysis, we were able to ensure a maximum contrast of functional cortical changes between two conditions (left‐side and right‐side) and thus investigate the behavior of this contrast as a surrogate of functional imaging capability during Valsalva‐induced eICP. In addition, a possible displacement of the activated volume during eICP (e.g., by minor shifts of the cortex or blood redistribution within the different arteriovenous compartments) as the explanation for the encountered amplitude changes could be made visible. However, corroborating the time course results, functional maps of the correct SI were obtained only during NB (Figure 7a,e,i,m). With parallel breathing maneuvers, no functional map could be recovered which makes a displacement of the origin of the Hb signal improbable. This finding applied to both, oxy‐Hb and deoxy‐Hb, which again is in contrast to our previous study that employed the same eICP paradigm on human MI during finger tapping and found a preserved oxy‐Hb functional map during eICP with an abolished functional imaging capability for deoxy‐Hb. When interpreting this disparity, the same points apply as in the discussion of the time course data. In summary, with regard to the sustained neuronal activity visible in the SEPs we encountered, we propose that deoxy‐Hb and oxy‐Hb do not indicate neuronal activation during eICP. This finding questions the validity of brain mapping based on these blood components in patients with eICP, for example, fMRI in brain tumor patients. However, the transfer of this study's results to patients with eICP cannot be done in a straightforward manner for two main reasons. Firstly, hypercapnia is a possible contributing factor in the applied VM but not in patients with eICP due to disease conditions. Secondly, as opposed to patients with disease‐related eICP, our paradigm only involved eICP transiently. Nevertheless, if this finding can be reproduced in future studies involving patients with chronic eICP, imaging methods based on deoxy‐Hb should be critically questioned toward their validity (Lindauer et al., 2010) and their ability to protect eloquent brain areas during surgery. Alternative imaging methods are, for example, navigated transcranial magnetic stimulation (Picht, Frey, Thieme, Kliesch, & Vajkoczy, 2016) or direct intraoperative electrical mapping (Kuchcinski et al., 2015) that do not depend on NVC. Our results also give reason to critically accompany the recent advance of fNIRS into the field of brain‐computer interfaces (Banville et al., 2017) since many applications rely on the presupposition of a measurable and valid NVC.

### 4.6 Perspective of a noninvasive ICP estimation

Our previous study with a gradual amplitude reduction of finger tapping induced deoxy‐Hb in the human MI during eICP gave rise to the outlook that this effect might be used to estimate ICP non‐invasively. Such a neuromonitoring method would be a relevant advance in neuromonitoring of neurocritical care patients. When deciding to do a parallel study during MNS we also wanted to transfer this paradigm to a functional activation method that is not dependent on cooperation and therefore can be applied to unconscious patients. Regarding the final results, we doubt that neuromonitoring by MNS will offer the possibility of ICP estimation. However, future studies should employ different, more robust stimulation paradigms, for example, visual stimulation, to further investigate this clinically relevant approach.

## 5 CONCLUSIONS

When applying eICP by escalating breathing maneuvers to human SI activation by MNS we encountered a significant reduction and even abolishment of both oxy‐Hb and deoxy‐Hb responses with preserved neuronal activity as measured by EEG. Thus, this study adds evidence to the hypothesis that eICP decouples Hb changes from the neuronal activity and therefore renders methods like BOLD‐fMRI and fNIRS blind to neuronal activation.

## CONFLICT OF INTEREST

The authors declare no potential conflicts of interest with respect to the research, authorship, and/or publication of this article.

## AUTHOR CONTRIBUTIONS

J.T.: conduction of experiments, data acquisition, analysis and interpretation of the data, drafting of the manuscript. M.K.: conduction of experiments, revisions of the manuscript. M.H.: conduction of experiments, revisions of the manuscript. J.K.: revisions of the manuscript. T.M.: revisions of the manuscript. G.R.: concept and design, data acquisition, analysis and interpretation of the data, drafting of the manuscript.

## Supporting information


**Figure S1** PetCO_2_ measurements obtained in additional measurements on 5 subjects performing randomized breathing maneuvers analogue to fNIRS experiments. Averaged across blocks, all subjects show a transient increase in PetCO_2_ following BH, V15, and V35 (a). Absolute and relative changes (b) indicate an absolute increase by 3–4 mmHg (9–10%) compared to baseline values in all breathing maneuvers except for NB (bef.: before, aft.: after, NC.: next condition, NB: normal breathing, BH: breath holding, V15: Valsalva maneuver with 15 mmHg forced expiratory pressure against resistance, V35: Valsalva maneuver with 35 mmHg forced expiratory pressure against resistance).

## Data Availability

The raw data that are included in this study are available from the corresponding author, upon reasonable request.

## References

[hbm24973-bib-0001] Aletti, F. , Re, R. , Pace, V. , Contini, D. , Molteni, E. , Cerutti, S. , … Baselli, G. (2012). Deep and surface hemodynamic signal from functional time resolved transcranial near infrared spectroscopy compared to skin flowmotion. Computers in Biology and Medicine, 42(3), 282–289. 10.1016/j.compbiomed.2011.06.001 21742320

[hbm24973-bib-0002] Backes, W. H. , Mess, W. H. , van Kranen‐Mastenbroek, V. , & Reulen, J. P. (2000). Somatosensory cortex responses to median nerve stimulation: FMRI effects of current amplitude and selective attention. Clinical Neurophysiology: Official Journal of the International Federation of Clinical Neurophysiology, 111(10), 1738–1744. 11018487 10.1016/s1388-2457(00)00420-x

[hbm24973-bib-0003] Banville, H. , Gupta, R. , & Falk, T. H. (2017). Mental task evaluation for hybrid NIRS‐EEG brain‐computer interfaces. Computational Intelligence and Neuroscience, 2017, 24 10.1155/2017/3524208 PMC5664195 29181021

[hbm24973-bib-0004] Blankenburg, F. , Taskin, B. , Ruben, J. , Moosmann, M. , Ritter, P. , Curio, G. , & Villringer, A. (2003). Imperceptible stimuli and sensory processing impediment. Science (New York, N.Y.), 299(5614), 1864 10.1126/science.1080806 12649475

[hbm24973-bib-0005] Brimioulle, S. , Moraine, J. J. , Norrenberg, D. , & Kahn, R. J. (1997). Effects of positioning and exercise on intracranial pressure in a neurosurgical intensive care unit. Physical Therapy, 77(12), 1682–1689. 9413447 10.1093/ptj/77.12.1682

[hbm24973-bib-0006] Buchner, H. , Claßen, J. , Curio, G. , Ferbert, A. , Haupt, W. F. , Hecht, M. , … Wessel, K. (2014). Praxisbuch evozierte Potenziale. Grundlagen, Befundung, Beurteilung und differentialdiagnostische Abgrenzung. Stuttgart: Thieme.

[hbm24973-bib-0007] Cope, M. , & Delpy, D. T. (1988). System for long‐term measurement of cerebral blood and tissue oxygenation on newborn infants by near infra‐red transillumination. Medical & Biological Engineering & Computing, 26(3), 289–294. 2855531 10.1007/BF02447083

[hbm24973-bib-0008] Füchtemeier, M. , Leithner, C. , Offenhauser, N. , Foddis, M. , Kohl‐Bareis, M. , Dirnagl, U. , … Royl, G. (2010). Elevating intracranial pressure reverses the decrease in deoxygenated hemoglobin and abolishes the post‐stimulus overshoot upon somatosensory activation in rats. NeuroImage, 52(2), 445–454. 10.1016/j.neuroimage.2010.04.237 20420930

[hbm24973-bib-0009] Handwerker, D. A. , Gazzaley, A. , Inglis, B. A. , & D'Esposito, M. (2007). Reducing vascular variability of fMRI data across aging populations using a breathholding task. Human Brain Mapping, 28(9), 846–859. 10.1002/hbm.20307 17094119 PMC6871393

[hbm24973-bib-0010] Henderson, L. A. , Macey, P. M. , Macey, K. E. , Frysinger, R. C. , Woo, M. A. , Harper, R. K. , … Harper, R. M. (2002). Brain responses associated with the Valsalva maneuver revealed by functional magnetic resonance imaging. Journal of Neurophysiology, 88(6), 3477–3486. 10.1152/jn.00107.2002 12466462

[hbm24973-bib-0011] Hillman, E. M. C. (2014). Coupling mechanism and significance of the BOLD signal: A status report. Annual Review of Neuroscience, 37, 161–181. 10.1146/annurev-neuro-071013-014111 PMC4147398 25032494

[hbm24973-bib-0012] Iadecola, C. (2017). The neurovascular unit coming of age: A journey through neurovascular coupling in health and disease. Neuron, 96(1), 17–42. 10.1016/j.neuron.2017.07.030 28957666 PMC5657612

[hbm24973-bib-0013] Kampe, K. K. , Jones, R. A. , & Auer, D. P. (2000). Frequency dependence of the functional MRI response after electrical median nerve stimulation. Human Brain Mapping, 9(2), 106–114. 10680767 10.1002/(SICI)1097-0193(200002)9:2<106::AID-HBM5>3.0.CO;2-Y PMC6871875

[hbm24973-bib-0014] Kamran, M. A. , Mannan, M. M. N. , & Jeong, M. Y. (2016). Cortical signal analysis and advances in functional near‐infrared spectroscopy signal: A review. Frontiers in Human Neuroscience, 10, 261 10.3389/fnhum.2016.00261 27375458 PMC4899446

[hbm24973-bib-0015] Kirilina, E. , Jelzow, A. , Heine, A. , Niessing, M. , Wabnitz, H. , Brühl, R. , … Tachtsidis, I. (2012). The physiological origin of task‐evoked systemic artefacts in functional near infrared spectroscopy. NeuroImage, 61(1), 70–81. 10.1016/j.neuroimage.2012.02.074 22426347 PMC3348501

[hbm24973-bib-0016] Klingner, C. M. , Huonker, R. , Flemming, S. , Hasler, C. , Brodoehl, S. , Preul, C. , … Witte, O. W. (2011). Functional deactivations: Multiple ipsilateral brain areas engaged in the processing of somatosensory information. Human Brain Mapping, 32(1), 127–140. 10.1002/hbm.21006 21157879 PMC6870510

[hbm24973-bib-0017] Knauth, M. , Heldmann, M. , Münte, T. F. , & Royl, G. (2017). Valsalva‐induced elevation of intracranial pressure selectively decouples deoxygenated hemoglobin concentration from neuronal activation and functional brain imaging capability. NeuroImage, 162, 151–161. 10.1016/j.neuroimage.2017.08.062 28860104

[hbm24973-bib-0018] Kuchcinski, G. , Mellerio, C. , Pallud, J. , Dezamis, E. , Turc, G. , Rigaux‐Viodé, O. , … Oppenheim, C. (2015). Three‐tesla functional MR language mapping: Comparison with direct cortical stimulation in gliomas. Neurology, 84(6), 560–568. 10.1212/WNL.0000000000001226 25589667

[hbm24973-bib-0019] Leithner, C. , & Royl, G. (2014). The oxygen paradox of neurovascular coupling. Journal of Cerebral Blood Flow and Metabolism: Official Journal of the International Society of Cerebral Blood Flow and Metabolism, 34(1), 19–29. 10.1038/jcbfm.2013.181 PMC3887356 24149931

[hbm24973-bib-0020] Lindauer, U. , Dirnagl, U. , Füchtemeier, M. , Böttiger, C. , Offenhauser, N. , Leithner, C. , & Royl, G. (2010). Pathophysiological interference with neurovascular coupling—When imaging based on hemoglobin might go blind. Frontiers in Neuroenergetics, 2, 25. 10.3389/fnene.2010.00025 PMC2955428 20953238

[hbm24973-bib-0021] Lindholm, P. , & Lundgren, C. E. G. (2006). Alveolar gas composition before and after maximal breath‐holds in competitive divers. Undersea & Hyperbaric Medicine: Journal of the Undersea and Hyperbaric Medical Society, Inc, 33(6), 463–467. 17274316

[hbm24973-bib-0022] Masamoto, K. , Hirase, H. , Yamada, K. , & Kanno, I. (2016). Neurovascular coupling‐what next? Progress in Brain Research, 225, 269–272. 10.1016/bs.pbr.2016.03.007 27130420

[hbm24973-bib-0023] Mathias, E. J. , Plank, M. J. , & David, T. (2017). A model of neurovascular coupling and the BOLD response PART II. Computer Methods in Biomechanics and Biomedical Engineering, 20(5), 519–529. 10.1080/10255842.2016.1255733 27832702

[hbm24973-bib-0024] Moriguchi, Y. , Noda, T. , Nakayashiki, K. , Takata, Y. , Setoyama, S. , Kawasaki, S. , … Hanakawa, T. (2017). Validation of brain‐derived signals in near‐infrared spectroscopy through multivoxel analysis of concurrent functional magnetic resonance imaging. Human Brain Mapping, 38(10), 5274–5291. 10.1002/hbm.23734 28722337 PMC6866983

[hbm24973-bib-0025] Niederhauser, B. D. , Rosenbaum, B. P. , Gore, J. C. , & Jarquin‐Valdivia, A. A. (2008). A functional near‐infrared spectroscopy study to detect activation of somatosensory cortex by peripheral nerve stimulation. Neurocritical Care, 9(1), 31–36. 10.1007/s12028-007-9022-2 17975711

[hbm24973-bib-0026] Nuwer, M. R. , Comi, G. , Emerson, R. , Fuglsang‐Frederiksen, A. , Guérit, J. M. , Hinrichs, H. , … Rappelsburger, P. (1998). IFCN standards for digital recording of clinical EEG. International Federation of Clinical Neurophysiology. Electroencephalography and Clinical Neurophysiology, 106(3), 259–261. 9743285 10.1016/s0013-4694(97)00106-5

[hbm24973-bib-0027] Obrig, H. (2002). *Nahinfrarotspektroskopie des Gehirns*. (PhD Thesis). Humboldt‐Universität zu Berlin, Medizinische Fakultät ‐ Universitätsklinikum Charité. 10.18452/13845

[hbm24973-bib-0028] Okamoto, M. , Dan, H. , Sakamoto, K. , Takeo, K. , Shimizu, K. , Kohno, S. , … Dan, I. (2004). Three‐dimensional probabilistic anatomical cranio‐cerebral correlation via the international 10‐20 system oriented for transcranial functional brain mapping. NeuroImage, 21(1), 99–111. 14741647 10.1016/j.neuroimage.2003.08.026

[hbm24973-bib-0029] Pak, R. W. , Hadjiabadi, D. H. , Senarathna, J. , Agarwal, S. , Thakor, N. V. , Pillai, J. J. , & Pathak, A. P. (2017). Implications of neurovascular uncoupling in functional magnetic resonance imaging (fMRI) of brain tumors. Journal of Cerebral Blood Flow and Metabolism: Official Journal of the International Society of Cerebral Blood Flow and Metabolism, 37(11), 3475–3487. 10.1177/0271678X17707398 PMC5669348 28492341

[hbm24973-bib-0030] Picht, T. , Frey, D. , Thieme, S. , Kliesch, S. , & Vajkoczy, P. (2016). Presurgical navigated TMS motor cortex mapping improves outcome in glioblastoma surgery: A controlled observational study. Journal of Neuro‐Oncology, 126(3), 535–543. 10.1007/s11060-015-1993-9 26566653

[hbm24973-bib-0031] Prabhakar, H. , Bithal, P. K. , Suri, A. , Rath, G. P. , & Dash, H. H. (2007). Intracranial pressure changes during Valsalva manoeuvre in patients undergoing a neuroendoscopic procedure. Minimally Invasive Neurosurgery, 50(2), 98–101. 10.1055/s-2007-982505 17674296

[hbm24973-bib-0032] Pstras, L. , Thomaseth, K. , Waniewski, J. , Balzani, I. , & Bellavere, F. (2016). The Valsalva manoeuvre: Physiology and clinical examples. Acta Physiologica (Oxford, England), 217(2), 103–119. 10.1111/apha.12639 26662857

[hbm24973-bib-0033] Roy, C. S. , & Sherrington, C. S. (1890). On the regulation of the blood‐supply of the brain. The Journal of Physiology, 11(1–2), 85–158. 10.1113/jphysiol.1890.sp000321 PMC1514242 16991945

[hbm24973-bib-0034] Sasse, S. A. , Berry, R. B. , Nguyen, T. K. , Light, R. W. , & Mahutte, C. K. (1996). Arterial blood gas changes during breath‐holding from functional residual capacity. Chest, 110(4), 958–964. 8874252 10.1378/chest.110.4.958

[hbm24973-bib-0035] Sato, H. , Fuchino, Y. , Kiguchi, M. , Katura, T. , Maki, A. , Yoro, T. , & Koizumi, H. (2005). Intersubject variability of near‐infrared spectroscopy signals during sensorimotor cortex activation. Journal of Biomedical Optics, 10(4), 44001 10.1117/1.1960907 16178635

[hbm24973-bib-0036] Sato, H. , Kiguchi, M. , Maki, A. , Fuchino, Y. , Obata, A. , Yoro, T. , & Koizumi, H. (2006). Within‐subject reproducibility of near‐infrared spectroscopy signals in sensorimotor activation after 6 months. Journal of Biomedical Optics, 11(1), 014021 10.1117/1.2166632 16526898

[hbm24973-bib-0037] Scholkmann, F. , Gerber, U. , Wolf, M. , & Wolf, U. (2013). End‐tidal CO2: An important parameter for a correct interpretation in functional brain studies using speech tasks. NeuroImage, 66, 71–79. 10.1016/j.neuroimage.2012.10.025 23099101

[hbm24973-bib-0038] Seddon, F. , Thacker, J. , Jurd, K. , & Loveman, G. (2014). Effects of Valsalva manoeuvres and the “CO2‐off” effect on cerebral blood flow. Diving and Hyperbaric Medicine, 44(4), 187–192. 25596831

[hbm24973-bib-0039] Spiegel, J. , Tintera, J. , Gawehn, J. , Stoeter, P. , & Treede, R. D. (1999). Functional MRI of human primary somatosensory and motor cortex during median nerve stimulation. Clinical Neurophysiology: Official Journal of the International Federation of Clinical Neurophysiology, 110(1), 47–52. 10348320 10.1016/s0168-5597(98)00043-4

[hbm24973-bib-0040] Steinmetz, H. , Fürst, G. , & Meyer, B. U. (1989). Craniocerebral topography within the international 10‐20 system. Electroencephalography and Clinical Neurophysiology, 72(6), 499–506. 2471619 10.1016/0013-4694(89)90227-7

[hbm24973-bib-0041] Strangman, G. , Franceschini, M. A. , & Boas, D. A. (2003). Factors affecting the accuracy of near‐infrared spectroscopy concentration calculations for focal changes in oxygenation parameters. NeuroImage, 18(4), 865–879. 12725763 10.1016/s1053-8119(03)00021-1

[hbm24973-bib-0042] Strojnik, M. , & Paez, G. (2013). Spectral dependence of absorption sensitivity on concentration of oxygenated hemoglobin: Pulse oximetry implications. Journal of Biomedical Optics, 18(10), 108001 10.1117/1.JBO.18.10.108001 24089256

[hbm24973-bib-0043] Tachtsidis, I. , & Scholkmann, F. (2016). False positives and false negatives in functional near‐infrared spectroscopy: Issues, challenges, and the way forward. Neurophotonics, 3(3), 031405 10.1117/1.NPh.3.3.031405 27054143 PMC4791590

[hbm24973-bib-0044] Takeuchi, M. , Hori, E. , Takamoto, K. , Tran, A. H. , Satoru, K. , Ishikawa, A. , … Nishijo, H. (2009). Brain cortical mapping by simultaneous recording of functional near infrared spectroscopy and electroencephalograms from the whole brain during right median nerve stimulation. Brain Topography, 22(3), 197–214. 10.1007/s10548-009-0109-2 19705276 PMC2749167

[hbm24973-bib-0045] Tanosaki, M. , Hoshi, Y. , Iguchi, Y. , Oikawa, Y. , Oda, I. , & Oda, M. (2001). Variation of temporal characteristics in human cerebral hemodynamic responses to electric median nerve stimulation: A near‐infrared spectroscopic study. Neuroscience Letters, 316(2), 75–78. 11742719 10.1016/s0304-3940(01)02372-2

[hbm24973-bib-0046] Thomason, M. E. , & Glover, G. H. (2008). Controlled inspiration depth reduces variance in breath‐holding‐induced BOLD signal. NeuroImage, 39(1), 206–214. 10.1016/j.neuroimage.2007.08.014 17905599 PMC2151095

[hbm24973-bib-0047] Towle, V. L. , Bolaños, J. , Suarez, D. , Tan, K. , Grzeszczuk, R. , Levin, D. N. , … Spire, J. P. (1993). The spatial location of EEG electrodes: Locating the best‐fitting sphere relative to cortical anatomy. Electroencephalography and Clinical Neurophysiology, 86(1), 1–6. 7678386 10.1016/0013-4694(93)90061-y

[hbm24973-bib-0048] Trepel, M. (2017). Neuroanatomie. Struktur und Funktion. (7. Auflage). München: Elsevier.

[hbm24973-bib-0049] Tsubaki, A. , Kojima, S. , Furusawa, A. A. , & Onishi, H. (2013). Effect of valsalva maneuver‐induced hemodynamic changes on brain near‐infrared spectroscopy measurements. Advances in Experimental Medicine and Biology, 789, 97–103. 10.1007/978-1-4614-7411-1_14 23852482

[hbm24973-bib-0050] Villringer, A. , & Dirnagl, U. (1995). Coupling of brain activity and cerebral blood flow: Basis of functional neuroimaging. Cerebrovascular and Brain Metabolism Reviews, 7(3), 240–276. 8519605

[hbm24973-bib-0051] Vogel, P. (2006). Kursbuch Klinische Neurophysiologie (Buch + DVD): EMG ‐ ENG ‐ Evozierte Potentiale (2nd ed.). Stuttgart: Thieme.

[hbm24973-bib-0052] Wang, L. , Chen, D. , Olson, J. , Ali, S. , Fan, T. , & Mao, H. (2012). Re‐examine tumor‐induced alterations in hemodynamic responses of BOLD fMRI: Implications in presurgical brain mapping. Acta Radiologica (Stockholm, Sweden: 1987), 53(7), 802–811. 10.1258/ar.2012.120118 22850572

[hbm24973-bib-0053] Whittaker, J. R. , Driver, I. D. , Bright, M. G. , & Murphy, K. (2016). The absolute CBF response to activation is preserved during elevated perfusion: Implications for neurovascular coupling measures. NeuroImage, 125, 198–207. 10.1016/j.neuroimage.2015.10.023 26477657 PMC4692513

[hbm24973-bib-0054] Williams, B. (1981). Simultaneous cerebral and spinal fluid pressure recordings. I. Technique, physiology, and normal results. Acta Neurochirurgica, 58(3–4), 167–185. 7315549 10.1007/BF01407124

[hbm24973-bib-0055] Wostyn, P. , Audenaert, K. , & De Deyn, P. P. (2009). The Valsalva maneuver and Alzheimer's disease: Is there a link? Current Alzheimer Research, 6(1), 59–68. 19199876 10.2174/156720509787313943

[hbm24973-bib-0056] Wu, P. , Bandettini, P. A. , Harper, R. M. , & Handwerker, D. A. (2015). Effects of thoracic pressure changes on MRI signals in the brain. Journal of Cerebral Blood Flow and Metabolism: Official Journal of the International Society of Cerebral Blood Flow and Metabolism, 35(6), 1024–1032. 10.1038/jcbfm.2015.20 PMC4640249 25712496

