# Supplementary figures and images for: Elevation of intracranial pressure affects the relationship between hemoglobin concentration and neuronal activation in human somatosensory cortex
Source: Hum Brain Mapp. 2020 Mar 4;41(10):2702–16. doi: 10.1002/hbm.24973 (PMC7294068; doi:10.1002/hbm.24973)

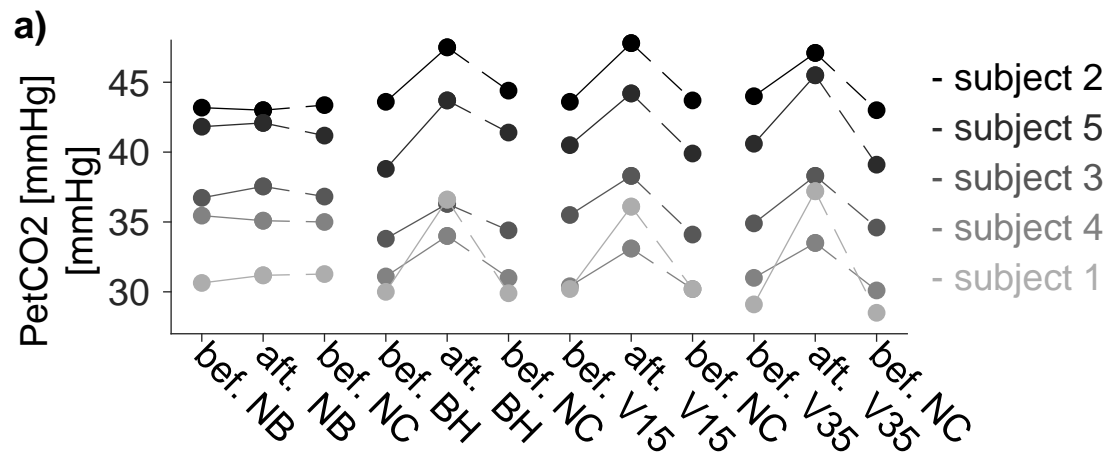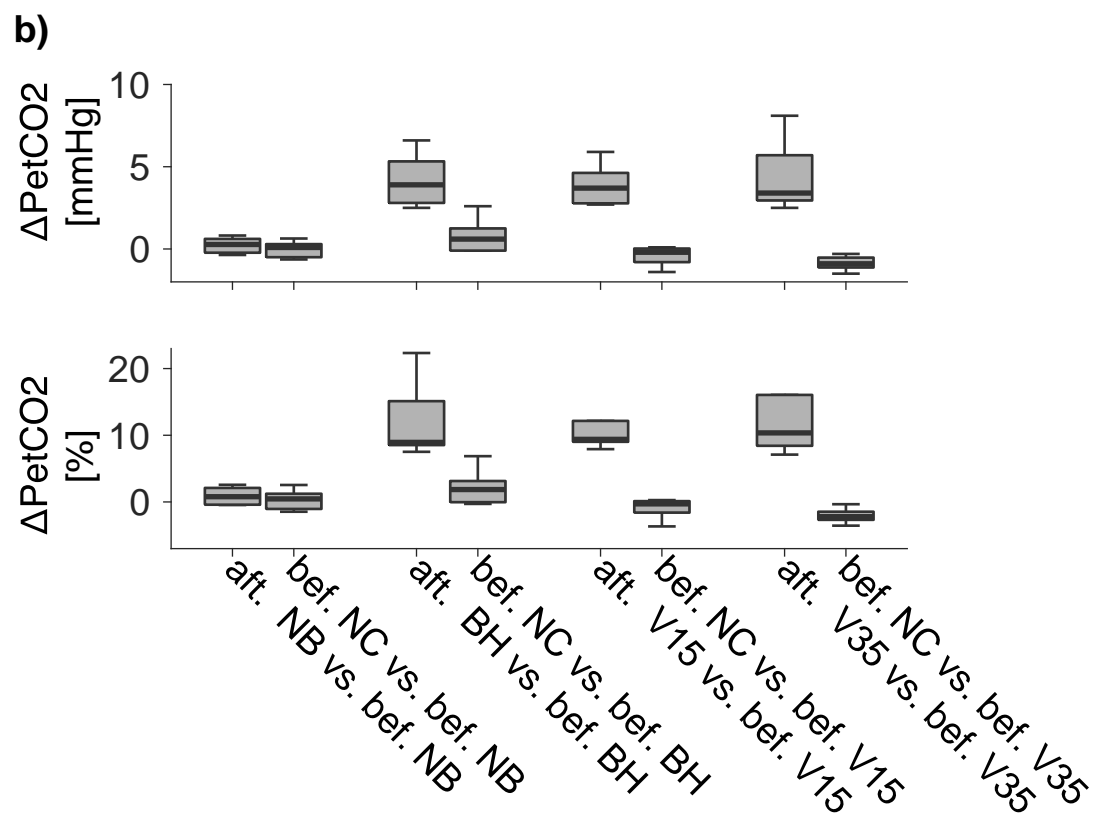

Supplement: Supplementary file 1 — Figure S1 PetCO2 measurements obtained in additional measurements on 5 subjects performing randomized breathing maneuvers analogue to fNIRS experiments. Averaged across blocks, all subjects show a transient increase in PetCO2 following BH, V15, and V35 (a). Absolute and relative changes (b) indicate an absolute increase by 3–4 mmHg (9–10%) compared to baseline values in all breathing maneuvers except for NB (bef.: before, aft.: after, NC.: next condition, NB: normal breathing, BH: breath holding, V15: Valsalva maneuver with 15 mmHg forced expiratory pressure against resistance, V35: Valsalva maneuver with 35 mmHg forced expiratory pressure against resistance). [file HBM-41-2702-s001.pdf]
